# Genetic alterations and protein expression of *KIT* and *PDGFRA* in serous ovarian carcinoma

**DOI:** 10.1038/sj.bjc.6602252

**Published:** 2004-12-07

**Authors:** H Lassus, H Sihto, A Leminen, S Nordling, H Joensuu, N N Nupponen, R Butzow

**Affiliations:** 1 Department of Obstetrics and Gynecology, Helsinki University Central Hospital, Haartmaninkatu 2, Helsinki 00290, Finland; 2 Department of Oncology, Helsinki University Central Hospital, Haartmaninkatu 4, Helsinki 00290, Finland; 3 Department of Pathology, University of Helsinki, PO Box 21 (Haartmaninkatu 3), Helsinki 00014, Finland

**Keywords:** ovarian neoplasms, cystadenocarcinoma, serous, KIT, PDGFRA, mutation

## Abstract

KIT and PDGFRA are receptor tyrosine kinases that can be specifically inactivated by small-molecule tyrosine kinase inhibitors, notably imatinib mesylate. In ovarian carcinoma, expression of KIT and PDGFRA protein has been documented, but the frequency and the molecular background of expression are poorly known. We analysed the expression of KIT and PDGFRA by immunohistochemistry in 522 serous ovarian carcinomas, and mutations of *KIT* and *PDGFRA* by denaturing high-performance liquid chromatographyin 125 and 187 serous ovarian carcinomas, respectively. No mutations of *KIT* or *PDGFRA* were detected. KIT expression was detected in 12% of carcinomas: low expression in 10% and high expression in 2% of cases. Using normal serous epithelium as a reference, decreased PDGFRA expression was detected in 12% and increased expression in 13% of carcinomas. Both KIT and PDGFRA expression were associated with high tumour grade, high proliferation index and poor patient outcome. By fluorescence *in situ* hybridisation, no *KIT* amplification was found in carcinomas with high KIT expression, but two cases showed a relative gain of chromosome 4. In conclusion, no mutations of *KIT* or *PDGFRA* were found, but a subset of serous ovarian carcinoma showed overexpression of the proteins, which was associated with aggressive tumour characteristics.

Most cases of ovarian carcinoma are disseminated at the time of the diagnosis requiring postoperative chemotherapy. Currently, combination chemotherapy using taxanes and platinum-based drugs is the standard treatment. Initially, majority of the patients respond to treatment, but ultimately over half of them experience disease recurrence and at this phase tumours are frequently resistant to currently used chemotherapy regimens. Thus, more effective treatment options against advanced ovarian carcinoma are needed.

KIT and PDGFRA belong to type III receptor tyrosine kinases, and they can be specifically targeted by tyrosine kinase inhibitors such as imatinib mesylate (STI571) (Fletcher, 2004). Somatic gain-of-function mutation of *KIT* has been documented in several human malignancies, notably acute myelogenous leukaemia, systemic mast cell disease, germ cell tumours and gastrointestinal stromal tumours (GIST) (Heinrich *et al*, 2002). KIT mutation has a key role in the pathogenesis of GISTs as demonstrated by the clinical effect of imatinib mesylate (Demetri *et al*, 2002). A subset of GISTs that lack *KIT* mutation harbour *PDGFRA* mutations and have shown response to imatinib therapy as well (Heinrich *et al*, 2003). In addition to point mutations, *PDGFRA* is activated by other mechanisms such as gene amplification in glioblastoma and chromosomal translocation leading to fusion protein formation in certain myeloproliferative diseases (Fletcher, 2004).

KIT and PDGFRA are not ubiquitous proteins, but their expression has also been reported in some epithelial malignancies including in ovarian carcinoma. This has raised hopes that some carcinomas could be treated with imatinib mesylate. In ovarian carcinomas, the reported frequency of KIT and PDGFRA expression has been highly variable, and little is known about their molecular background and association with clinical parameters (Henriksen *et al*, 1993; Inoue *et al*, 1994; Arber *et al*, 1998; Dabrow *et al*, 1998; Parrott *et al*, 2000; Tonary *et al*, 2000; Schmandt *et al*, 2003; Singer *et al*, 2003; Apte *et al*, 2004a; Matei *et al*, 2004). GISTs with KIT mutation, particularly in exon 11, show a clearly better response to imatinib therapy as compared to tumours with no mutation, suggesting that detection of gain-of-function mutation and not solely KIT expression should be a requirement for the treatment (Heinrich *et al*, 2003).

Ovarian carcinoma is a heterogeneous disease as regards tumour histology. In previous literature, different histological types, mainly serous, mucinous and endometrioid, have been treated as a single entity, but lately they have been shown to differ in their clinicopathological characteristics (Makar *et al*, 1995; Risch *et al*, 1996; Heintz *et al*, 2001) and molecular alterations (Klemi *et al*, 1995; Tapper *et al*, 1997; Obata *et al*, 1998; Lassus *et al*, 2001; Schwartz *et al*, 2002). In this study, we have concentrated on one histological type, serous carcinoma, which is the most common subtype of ovarian carcinoma and shows aggressive behaviour and secondary resistance to currently used adjuvant therapy.

To evaluate the molecular basis for use of imatinib mesylate in ovarian carcinoma, we analysed the frequency of *KIT* and *PDGFRA* mutations by denaturing high-performance liquid chromatography (DHPLC) and direct sequencing of aberrant exons in 125 and 187 serous ovarian carcinoma specimens, respectively. Protein expression status of KIT and PDGFRA was performed by immunohistochemistry of tissue microarray containing 522 serous ovarian carcinomas. Tumours showing aberrantly high expression of KIT were further tested for *KIT* amplification by fluorescence *in situ* hybridisation (FISH). The findings were correlated with clinicopathological and other molecular characteristics of the tumours and outcome of the patients.

## MATERIALS AND METHODS

### Mutation analysis

Tumour samples were obtained from patients undergoing primary surgery for ovarian carcinoma at the Department of Obstetrics and Gynecology, Helsinki University Central Hospital. Tumours with serous histology and tumour cell percentage over 60 (range 60–95, median 75%) were included in the study. Borderline tumours were excluded from the study, but otherwise the cases were not selected for stage or grade. *KIT* mutation analysis was performed from 111 fresh–frozen and from 14 paraffin-embedded samples, and *PDGFRA* mutation analysis from 187 fresh–frozen tumour samples. DNA was extracted from tumour tissue block after mechanical disruption directly (fresh–frozen samples) or after xylene extraction (paraffin-embedded samples). A standard proteinase-K–phenol–chloroform method was used for DNA extraction.

### PCR conditions for mutational analysis

Exons of 9, 11, 13 and 17 of *KIT* and exons 11 and 17 of *PDGFRA* (according to the Human Genome Project available at http://www.ensembl.org; exons 11 and 17 correspond to PDGFRA exons 12 and 18 of GenBank Accession number D50013, http://www.ncbi.nlm.nih.gov:80 /entrez/) were amplified from tumour samples using primers given in Table 1

**Table 1:** Primers used for PCR of exons of 9, 11, 13 and 17 of *KIT* and exons 11 and 17 of *PDGFRA*

**Exon**	**Forward primer**	**Reverse primer**
*KIT*		
9	GTATGCCACATCCCAAGTGT	CATGACTGATATGGTAGACA
11	CCAGAGTGCTCTAATGACTG	GGAAGCCACTGGAGTTCCTT
13	GACATCAGTTTGCCAGTTGT	TGTTTTGATAACCTGACAGAC
17	GCAACACTATAGTATTAAAAAG	CCTTTGCAGGACTGTCAAGCA
		
*PDGFRA*		
11	ATGTGGAGTGAACGTTGTTGG	CTAGTTCTTACTAAGCACAAGC
17	CAGGGGTGATGCTATTCAGC	TTAAAGTGAAGGAGGATGAGCC

. PCR was performed in 50 *μ*l reactions consisting of 20–50 ng genomic DNA, 0.2 mM dNTPs, 1 × PCR buffer (Gibco BRL, Gaithersburg, MD), 4.5 mM MgCl_2_, 0.5 U Platinum Taq DNA polymerase (Gibco BRL), 0.5 U AmpliTaq Gold (Applied Biosystems) and 10 pmol of each forward and reverse primer (Gibco BRL). PCR cycles consisted of 95°C for 14 min, followed by 35 cycles of 30 s at 95°C, 30 s at 55°C for exons of *KIT*/59°C for exons of *PDGFRA*, 45 s at 72°C and a final extension at 72°C for 5 min. Subsequently, the PCR fragments were analysed with DHPLC.

### Denaturing high-performance liquid chromatography

PCR products were denatured for 3 min at 95°C and then reannealed gradually over 30 min using a 95°C to 40°C temperature gradient. The optimal melting temperature for each PCR amplicon was obtained by analysis of the wild-type sequence, using an algorithm at the Stanford Denaturing High-Performance Liquid Chromatography website (http://insertion.stanford.edu/ melt.html). DHPLC heteroduplex analysis was performed using an automated HPLC instrumentation (Agilent Technologies) equipped with a Helix DNA column (Varian Inc., Netherlands). The analytical gradient was composed of Buffer A (100 mM triethylammonium acetate and 0.10 mM EDTA) and Buffer B (100 mM triethylammonium acetate, 0.10 mM EDTA and 25% acetonitrile) with a flow rate of 0.450 ml min^−1^. The injection volume of each PCR sample was 4–7 *μ*l. The analysis time for each sample was 7 min, including a short column wash and an equilibration step. GISTs harbouring mutations in exons 9, 11 or 17 (*KIT*) and 11 or 17 (*PDGFRA*) were used as positive controls for DHPLC analysis.

### Protein expression analysis by immunohistochemistry

#### Material and tissue microarray construction

Material for protein expression analysis consisted of 522 serous ovarian carcinomas, which have been characterised previously (Lassus *et al*, 2003). Tissue microarrays were constructed as described previously (Kononen *et al*, 1998). Tissue specimens from 34 normal ovarian and 23 normal fallopian tube samples and 522 serous ovarian carcinomas were arranged in six recipient paraffin blocks. Four core tissue biopsies were obtained from each specimen (Lassus *et al*, 2003).

#### Immunohistochemistry

Sections (5 *μ*m thick) were cut from each block on coated slides. The sections were deparaffinised in xylene and rehydrated through graded concentrations of ethanol. The slides were pretreated with citrate buffer (pH 6.0) in a microwave oven for 2 × 4 min and then cooled for 30 min before starting the staining procedure, which was performed for KIT in Dako Autostainer with the Envision System and for PDGFRA in Lab Vision Autostainer with the Ultravision System. The primary antibodies used were a polyclonal antibody against KIT (dilution 1 : 100; Dako, Carpinteria, CA, USA) and a polyclonal antibody against synthetic peptide derived from C-terminal of PDGFRA (dilution 1 : 150, Neomarkers, Lab Vision, Fremont, CA, USA). Negative controls were performed by replacing the primary antibody by normal rabbit serum. For KIT paraffin-embedded KIT-positive GISTs were used as positive controls.

The immunohistochemical analysis was evaluated by a pathologist (RB) without knowledge of the clinicopathological information. The epithelium of fallopian tubes (the normal serous epithelium of Müllerian origin) and ovarian surface epithelium (OSE) were used as a reference of normal expression for both proteins. Both membrane and cytoplasmic staining were taken into account and scored according to the intensity as follows: KIT – negative, weak or strong; and PDGFRA – weak, moderate or strong.

Immunohistochemistry for p53 and Ki-67 was performed as described previously (Lassus *et al*, 2004). A polyclonal antibody against Ki-67 (1 : 150, clone N/A; code A0047; Dako A/S, Glostrup, Denmark) and a monoclonal antibody against p53 (1 : 100; clone DO-7; Dako A/S, Glostrup, Denmark) were used as primary antibodies.

### Fluorescence *in situ* hybridisation

Paraffin-embedded samples of tumours showing distinct, strong KIT staining by immunohistochemistry were included in the FISH analysis.

Chromosome 4 was studied with a centromere-specific probe (CEP4 Spectrum Green, Vysis Inc., North Chicago, IL), and *KIT* gene with BAC probes (clones RP11-1106L19 and RP11-977G3). The correct probe identities were confirmed using PCR with the *KIT*-specific primers. The BAC-DNAs were isolated using routine techniques and labelled with DIG-Nick translation mix (Roche, Mannheim, Germany). The dual-colour hybridisations were performed as described previously (Hyytinen *et al*, 1994). The digoxigenin-labelled probes were detected by sheep anti-digoxigenin–rhodamine antibody (Roche, Mannheim, Germany).

Interphase nuclei were prepared as described previously (Hyytinen *et al*, 1994). *KIT* probe and chromosome 4 centromere probe were cohybridised and after hybridisation, counterstained with DAPI and viewed under a fluorescence microscopy equipped with ISIS digital image analysis system (MetaSystems). Approximately 50 interphase nuclei were analysed of each sample and percentages/subpopulation were calculated for normal and abnormal nuclei.

### DNA ploidy analysis

Core tissue biopsy specimen (diameter 0.8 mm) were taken from areas representing carcinoma in paraffin tissue block. The tissue cores were deparaffinised, rehydrated and DNA flow cytometry was performed as described previously (Jahkola *et al*, 1998).

### Statistical analysis

Associations between factors were analysed with the *χ*^2^ and Fisher's exact tests. The overall and disease-free survival curves were constructed according to the Kaplan–Meier method and compared with the log-rank test. For multivariate survival analysis Cox's proportional-hazards model was used, with backward stepwise selection procedure, and entering the following as categorial covariates: FIGO stage (stages I, II, III and IV), grade (grades 1, 2 and 3), age at diagnosis (<57 years (median) and ⩾57 years), tumour size (⩽10 cm and >10 cm), residual tumour size (⩽1 cm and >1 cm), ascites (presence or absence), p53 expression (normal and aberrant) and Ki-67 expression (0–10, 10–25 and >25%). A *P*-value of 0.05 was adopted as the limit for inclusion of a covariate. All *P*-values are two-sided.

## RESULTS

### Mutation analysis of *KIT* and *PDGFRA*

In DNA from freshly frozen tissue samples, DHPLC analysis of at least one exon failed in approximately 5% of cases due to poor amplification of the sample DNA. Out of 14 paraffin-embedded tumours, 11 were successfully analysed. In cases of aberrant DHPLC profile, the analysis was repeated and doubtful cases were sequenced. For *KIT* analysis, exons 9 and 13 were sequenced in two samples. For *PDGFRA* analysis, exon 17 was sequenced in one sample and exon 11 in five samples. No sequence alterations were found.

### KIT expression and clinicopathological associations

**Figure 1:**
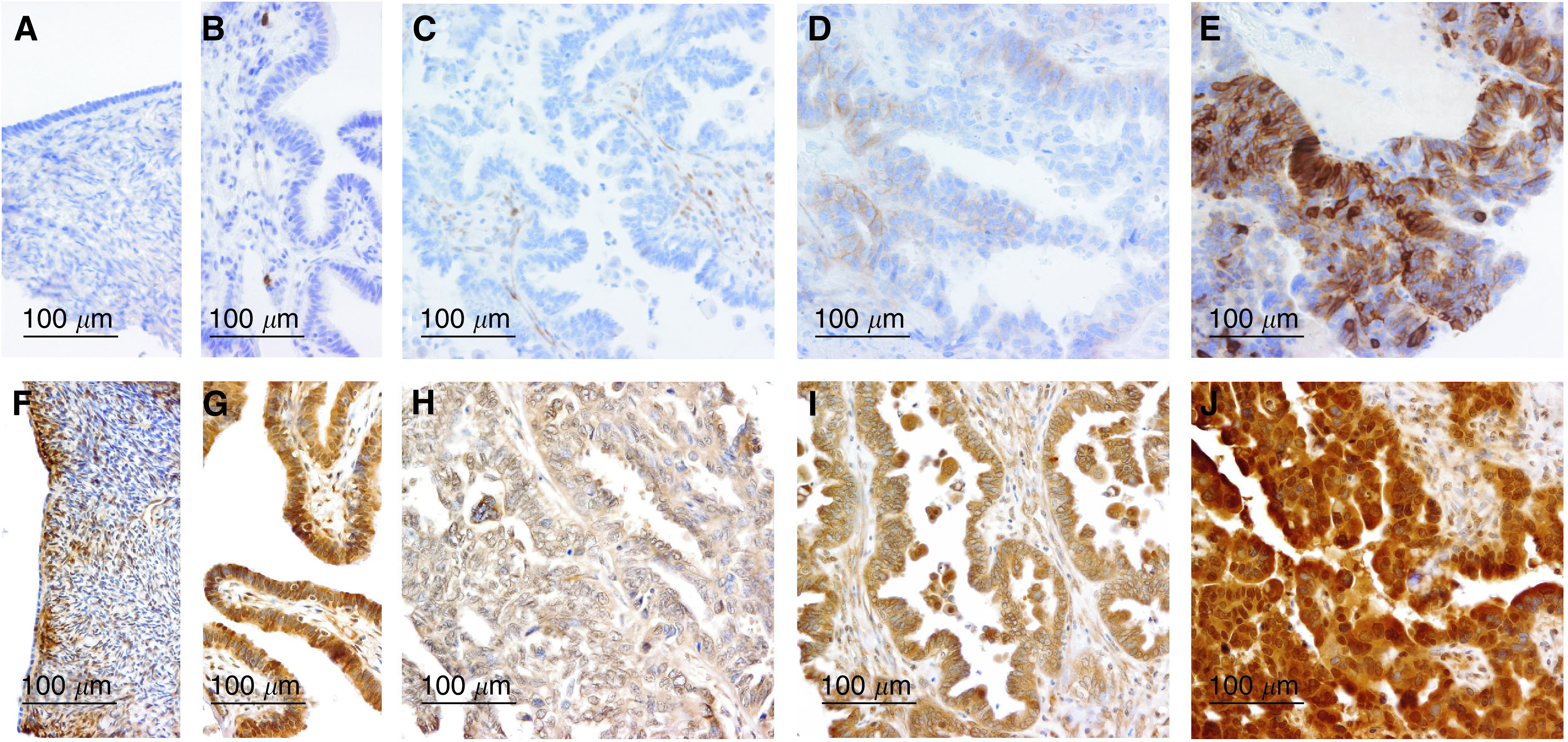
Examples of KIT and PDGFRA expression by immunohistochemistry. Normal ovarian surface (**A**) and tubal (**B**) epithelium showing negative immunostaining of KIT protein. Serous ovarian carcinomas showing negative (**C**), weak (**D**) and strong (**E**) staining of KIT protein. Normal OSE showing negative immunostaining of PDGFRA (**F**) and tubal epithelium showing moderate immunopositivity of PDGFRA (**G**). Serous ovarian carcinomas showing weak (**H**), moderate (**I**) and strong (**J**) staining of PDGFRA.

The epithelium of normal ovarian surface and fallopian tubes was negative for KIT protein (Figure 1A and B

). In the stroma of fallopian tubes, there were single cells with strong cytoplasmic staining (Figure 1B), compatible with mast cells. The stromal cells of normal ovarian cortex showed variable, mainly weak, positivity (Figure 1A). KIT immunostaining was interpretable in 516 (99%) of the 522 serous ovarian carcinomas. No staining (negative) was detected in 453 (88%), weak positive immunostaining in 51 (9.9%) and strong positive immunostaining in 12 (2.3%) of the interpretable cases (Figure 1C–E).

KIT expression was associated with high tumour grade (*P*<0.0001), advanced age (*P*=0.0198), high proliferation index (*P*=0.0004) and aberrant p53 status (*P*=0.0053), but not with tumour stage, tumour size, residual tumour size or the presence of ascites (Table 2

**Table 2:** Association of KIT expression with clinicopathological characteristics

	**KIT immunohistochemical score**						
**Clinicopathological characteristics**	**Negative**	**%**	**Weak positive**	**%**	**Strong positive**	**%**	***P*-value**
*FIGO stage*							
I	100/108	93	5/108	4	3/108	3	NS; 0.39
II	56/64	87	8/64	13	0/64	0	
III	243/281	86	31/281	11	7/281	2	
IV	51/60	85	7/60	12	2/60	3	
							
*Grade*							
1	186/194	96	5/194	3	3/194	2	<0.0001
2	110/132	83	20/132	15	2/132	2	
3	150/182	82	25/182	14	7/182	4	
							
*Residual tumour*							
⩽1 cm	201/236	85	27/236	11	8/236	3	NS; 0.23
>1 cm	204/228	89	21/228	9	3/228	1	
							
*Age*							
⩽57 years	229/249	92	16/249	6	4/249	2	0.0198
>57 years	224/267	84	35/267	13	8/267	3	
							
*Tumour size*							
⩽10 cm	158/179	88	16/179	9	5/179	3	NS; 0.83
>10 cm	287/327	88	33//327	10	7/327	2	
							
*Ascites*							
No	168/185	91	14/185	8	3/185	2	NS; 0.29
Yes	277/322	86	37/322	11	8/322	2	
							
*Ki-67*							
Low (0–10%)	253/270	94	14/270	5	3/270	1	0.0004
Moderate (10–25%)	106/130	82	19/130	15	5/130	4	
High (>25%)	80/101	79	17/101	17	4/101	4	
							
*p53*							
Normal	192/206	93	10/206	5	4/206	2	0.0053
Aberrant	248/295	84	40/295	14	7/295	2	

NS=not significant.

).

**Figure 2:**
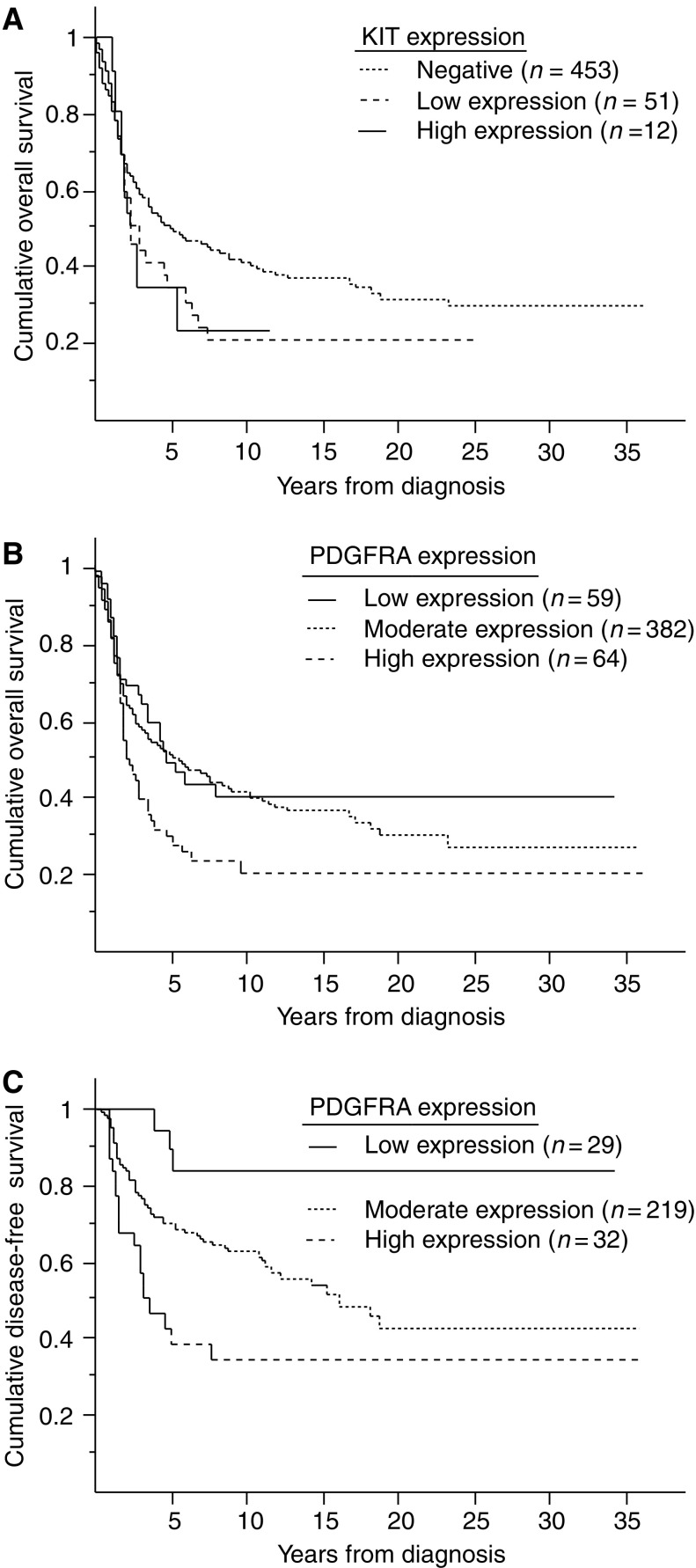
Overall survival in patients with serous ovarian carcinoma in relation to KIT expression (**A**) and PDGFRA expression (**B**) by immunohistochemistry. Disease-free survival in patients with serous ovarian carcinoma in relation to PDGFRA expression (**C**).

Patients with tumours showing KIT expression (low or high) were associated with shorter overall survival compared with cases showing negative KIT expression (Figure 2A

). The 5-year overall survival rates for patients with tumours with negative, weak positive and strong positive KIT expression were 50% (95% CI, 45–55%), 34% (19–50%) and 35% (3–66%), respectively. When tumours with low and high KIT expression were analysed as one group (positive KIT expression), the difference was statistically significant (*P*=0.0414). There was also a tendency for a shorter disease-free survival in patients with positive KIT expression, but the association was not statistically significant (*P*=0.0875).

### PDGFRA expression and clinicopathological associations

The epithelium of normal ovarian surface was negative for PDGFRA expression (Figure 1F), whereas the serous epithelium of fallopian tubes showed variable, moderate immunopositivity (Figure 1G). PDGFRA immunostaining was interpretable in 505 (97%) of the 522 serous ovarian carcinomas. Weak positive immunostaining was detected in 59 (12%), moderate immunostaining, corresponding to that of fallopian tube epithelium, in 382 (75%) and strong immunostaining in 64 (13%) of the interpretable cases (Figure 1H–J).

Strong PDGFRA staining was associated with high tumour grade (*P*=0.0019), high tumour stage (*P*=0.0483), large residual tumour size (*P*=0.0330) and high proliferation index (*P*=0.0060), but not with advanced age, tumour size, presence of ascites or p53 status (Table 3

**Table 3:** Association of PDGFRA expression with clinicopathological characteristics

	**PDGFRA immunohistochemical score**						
**Clinicopathological characteristics**	**Weak**	**%**	**Moderate**	**%**	**Strong**	**%**	***P*-value**
*FIGO stage*							
I	12/58	21	83/380	22	9/64	14	0.0483
II	12/58	21	44/380	12	10/64	16	
III	23/58	40	208/380	55	41/64	64	
IV	11/58	19	45/380	12	4/64	6	
							
*Grade*							
1	22/59	37	153/375	41	13/63	21	0.0019
2	11/59	19	103/375	27	15/63	24	
3	26/59	44	119/375	32	35/63	56	
							
*Residual tumour*							
⩽1 cm	24/49	49	178/345	51	20/60	33	0.0330
>1 cm	25/49	51	167/345	49	40/60	67	
							
*Age*							
<57 years	29/59	49	185/382	48	31/64	48	NS; 0.99
⩾57 years	30/59	51	197/382	52	33/64	52	
							
*Tumour size*							
⩽10 cm	26/58	45	122/377	32	23/61	38	NS; 0.15
>10 cm	32/58	55	255/377	68	38/61	62	
							
*Ascites*							
No	26/56	46	130/377	34	22/63	35	NS; 0.22
Yes	30/56	54	247/377	66	41/63	65	
							
*Ki-67*							
0–10%	39/57	68	200/375	53	24/62	39	0.0060
10–25%	9/57	16	105/375	28	17/62	27	
>25%	9/57	16	70/375	19	21/62	34	
							
*p53*							
Normal	27/58	47	155/372	42	17/63	27	NS; 0.053
Aberrant	31/58	53	217/372	58	46/63	73	

NS=not significant.

). PDGFRA expression was not associated with KIT expression (*P*=0.13).

High PDGFRA expression was associated with shorter overall survival (*P*=0.0353) (Figure 2B). The 5-year overall survival rates for patients with tumours with low, moderate and high PDGFRA expression were 49% (95% CI, 34–63%), 51% (46–57%) and 31% (19–43%), respectively. PDGFRA expression was also associated with disease-free survival (*P*=0.0003) (Figure 2C). The 5-year disease-free survival rates for patients with tumours with low, moderate and high PDGFRA expression were 90% (95% CI, 76–100%), 70% (64–77%) and 39% (21–57%), respectively.

### *KIT* and chromosome 4 copy number by FISH

**Figure 3:**
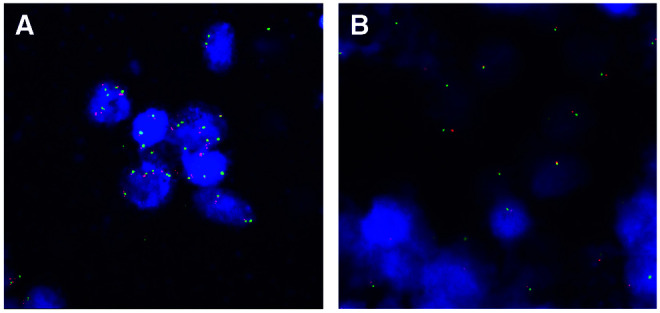
Examples of copy number analysis of *KIT* gene and chromosome 4 centromere by FISH: serous ovarian carcinomas showing a subpopulation of cells with seven signals for both probes (case 2283) (**A**) and a loss of other chromosome 4 and *KIT* gene (case 1029) (**B**).

FISH analysis was successful in 10 out of 12 tumours showing high KIT expression. Six of these 10 tumours showed a normal copy number (two signals) for both chromosome 4 centromere probe and the *KIT* probe. One tumour revealed a subpopulation of nuclei with tetrasomy, one tumour had a subpopulation of nuclei with five FISH-signals for both probes and one tumour showed a subpopulation with seven FISH signals for both probes (Figure 3A

). One tumour showed a loss of other chromosome 4 and *KIT* gene (Figure 3B). No high-level amplification was observed in any of the tumours analysed. The results of FISH and ploidy analysis of these tumours are shown in Table 4

**Table 4:** Copy number of KIT and chromosome 4, ploidy, expression of Ki-67 and p53 in serous ovarian carcinomas showing high expression of KIT protein

**Case**	***KIT***	**Chr. 4**	**Ploidy**	**Ki-67**	**p53**
7	2	2	Diploid	Moderate	Normal
489	2	2	45% diploid, 55% hyperdiploid (DI=1.45)	High	Aberrant
859	2/5	2/5	Diploid	High	Aberrant
1029	1	1	Diploid	High	Normal
1164	2	2	Diploid	High	Aberrant
2013	2	2	Diploid	Low	Normal
2120	2/4	2/4	46% diploid, 54% hyperdiploid (DI=1.84)	Moderate	Aberrant
2175	NI	NI	50% hyperdiploid (DI=1.5), 50% hypertetraploid (DI=3)	Low	NI
2280	2	2	Hypertetraploid (DI=2.19)	Moderate	Aberrant
2283	2/7	2/7	Hyperdiploid (DI=1.73)	Moderate	Aberrant
2381	NI	NI	Diploid	Moderate	Aberrant
2636	2	2	Diploid	Low	Normal

NI=not informative; DI=DNA index; categories: 1–1.2=diploid, 1.21–1.90=hyperdiploid, 1.91–2.1=tetraploid, 2.11–=hypertetraploid.

.

## DISCUSSION

No *KIT* or *PDGFRA* mutations were found in serous ovarian carcinomas. In our analysis, we concentrated on the juxtamembrane and catalytic domains, that is, exons 9, 11, 13 and 17 of *KIT* and exons 11 and 17 of *PDGFRA*, where the activating mutations in *KIT* and *PDGFRA* have been detected (Heinrich *et al*, 2002; Heinrich *et al*, 2003). As regards *KIT*, our finding extends the previous observation of no mutations in 50 ovarian carcinomas of different histological types (Singer *et al*, 2003). As regards *PDGFRA*, to our knowledge, no previous reports on ovarian carcinoma exist in the literature.

KIT expression in normal ovaries and ovarian carcinomas has been addressed in several previous studies with highly variable results. At least one study (Parrott *et al*, 2000) reported KIT immunoreactivity in normal OSE, but most reports do not confirm this finding (Inoue *et al*, 1994; Tonary *et al*, 2000; Schmandt *et al*, 2003; Singer *et al*, 2003). In our analysis both OSE and fallopian tube epithelium, the normal serous epithelium of Müllerian origin, were negative for KIT protein. Many reports have indicated high frequency (71–100%) of KIT expression in ovarian carcinomas (Arber *et al*, 1998; Parrott *et al*, 2000; Tonary *et al*, 2000), whereas others have shown lower levels of expression (0–22%) (Inoue *et al*, 1994; Schmandt *et al*, 2003; Singer *et al*, 2003). We found the expression of KIT protein in 12% of serous ovarian carcinomas: 10% showed low and 2% high expression of the protein. Disparities in the findings of different studies may reflect heterogeneity in the study material as regards tumour histology and other clinicopathological parameters. More importantly, different antibodies and staining protocols have been used. Our findings are closest to those reported by Schmandt *et al* (2003) and Singer *et al* (2003), who also used the KIT CD117 polyclonal antibody (Dako), which is accepted for clinical use while assessing the KIT expression in GIST (Fletcher *et al*, 2002). We used the same antibody dilution and IHC protocol as routinely used in GIST diagnostics. Weak expression was found to be mainly cytoplasmic, whereas in strongly positive cases, membranous staining was also detected, presumably representing the active form of KIT protein (Tian *et al*, 1999; Shaw *et al*, 2002).

KIT expression was associated with poor histological differentiation, high patient age and poor patient outcome. The association of KIT expression with poor clinical outcome is in line with the presumed oncogenic properties of KIT. Schmandt *et al* (2003) also reported association of KIT expression with high tumour grade but contradictory findings exist. Tonary *et al* (2000) reported KIT expression to be independent of tumour grade, but associated with low tumour stage and favourable patient outcome. In that particular study, the frequency of KIT expressing tumours was very high (71%), indicating differences in the methodology and material employed. In our study, tumours with KIT expression more often presented with high proliferation index and aberrant p53 status. Interestingly, the associations of KIT expression with grade, age, Ki-67 and p53 were independent of the degree of KIT expression (low or high). The association of KIT with high growth fraction is consistent with presumed proliferation promoting effect of KIT. However, in ovarian carcinoma cell lines, KIT inhibition by anti-KIT neutralising antibodies or the KIT inhibitor STI571 did not alter the growth rate (Shaw *et al*, 2002). The real biological role of KIT in ovarian carcinoma cells remains to be clarified.

The normal OSE did not express PDGFRA protein, which is in line with previous findings (Henriksen *et al*, 1993). However, the epithelium of fallopian tubes showed variable, moderate immunopositivity, and similar expression pattern was seen in majority (75%) of the carcinomas. Using this as a reference, 12% of serous ovarian carcinomas showed decreased and 13% increased PDGFRA expression. In previous reports, PDGFRA immunopositivity has varied from 5 to 100% of ovarian carcinomas (Henriksen *et al*, 1993; Dabrow *et al*, 1998; Apte *et al*, 2004a; Matei *et al*, 2004). Little is known about its associations with clinical parameters. Henriksen *et al* (1993) also found PDGFRA positivity to associate with poor overall survival. In our study, the association with disease-free survival was even stronger than that with overall survival. In line with aggressive tumour behaviour, PDGFRA expression was also associated with high tumour grade and stage, large residual tumour size and high proliferation index.

In all, 12 carcinomas presented with distinct KIT overexpression and amplification of *KIT* gene was considered as a possible mechanism for overexpression. However, FISH analysis revealed no gene amplification. Six tumours out of 10 showed a normal copy number, three showed polysomy and one monosomy of chromosome 4. In two tumours showing five and seven copies of chromosome 4, the tumour cells were diploid/hyperdiploid, indicating a relative gain of chromosome 4. In two cases, there was a relative loss of chromosome 4: one with monosomy of chromosome 4 and diploid DNA and the other with two copies of chromosome 4 and hypertetraploid DNA. *KIT* gene is located at the proximal part of chromosome arm 4q (4q11–12). According to cytogenetic and comparative genomic hybridisation studies, gain of chromosome 4 is a very rare event in ovarian carcinoma. The relative gain of chromosome 4 we observed in two out of 10 tumours is an unexpected finding (http://cgap.nci.nih.gov/Chromo somes/RecurrentAberrations; http://ethesis.helsinki.fi/jul kaisut/laa/kliin/vk/lassus/) and may account for the overexpression of KIT in these cases. Interstitial chromosomal deletion on 4q12 yielding active fusion protein FIPIL1-PDGFRA plays a causal role in a portion of idiopathic hypereosinophilia syndrome and chronic eosinophilic leukaemia cases that can successfully be treated with imatinib mesylate (Coutre and Gotlib, 2004). Interestingly, loss of chromosomal material from 4q is frequent in serous ovarian carcinoma (reviewed in http://ethesis.helsinki.fi/jul kaisut/laa/kliin/vk/lassus/) and gain-of-function deletion is an intriguing alternative mechanism for PDGFRA overexpression.

The lack of *KIT* and *PDGFRA* mutations seems discouraging as regards potential usefulness of imatinib mesylate in serous ovarian carcinoma. In GISTs (*KIT* and *PDGFRA* mutations), breast carcinoma (HER-2 amplification) and lung cancer (EGFR mutation), targeted therapy has yielded best results in cases with activating mutation or amplification of the respective gene (Vogel *et al*, 2002; Heinrich *et al*, 2003; Lynch *et al*, 2004). However, in our study, both KIT and PDGFRA expression were associated with aggressive tumour characteristics, such as high tumour grade, high proliferation index and poor patient outcome, suggesting them a role in the pathophysiology of at least a subset of serous ovarian carcinomas. Accordingly, imatinib mesylate has inhibited growth of ovarian cancer cells through PDGFRA and Akt inactivation (Matei *et al*, 2004), and combination therapy of imatinib–paclitaxel has impaired progression of ovarian cancer in peritoneal cavity of nude mice and lead to increased apoptosis of tumour-associated endothelial cells (Apte *et al*, 2004b). The possible usefulness of imatinib mesylate in the treatment of ovarian carcinoma can only be resolved in clinical trials. If such were to be conducted, KIT or PDGFRA overexpression, and not mutational status of the genes, would seem as appropriate criteria for selection of patients.
